# Effects of brain polarization on reaction times and pinch force in chronic stroke

**DOI:** 10.1186/1471-2202-7-73

**Published:** 2006-11-03

**Authors:** Friedhelm C Hummel, Bernhard Voller, Pablo Celnik, Agnes Floel, Pascal Giraux, Christian Gerloff, Leonardo G Cohen

**Affiliations:** 1 Human Cortical Physiology Section and Stroke Neurorehabilitation Clinic, National Institute of Neurological Disorders and Stroke, National Institutes of Health, Bethesda, MD 20892, USA; 2 Brain Imaging and Neurostimulation Lab, Department of Neurology, University Medical Center Hamburg-Eppendorf, Martinistr. 52, 20246 Hamburg, Germany

## Abstract

**Background:**

Previous studies showed that anodal transcranial DC stimulation (tDCS) applied to the primary motor cortex of the affected hemisphere (M1_affected hemisphere_) after subcortical stroke transiently improves performance of complex tasks that mimic activities of daily living (ADL). It is not known if relatively simpler motor tasks are similarly affected. Here we tested the effects of tDCS on pinch force (PF) and simple reaction time (RT) tasks in patients with chronic stroke in a double-blind cross-over Sham-controlled experimental design.

**Results:**

Anodal tDCS shortened reaction times and improved pinch force in the paretic hand relative to Sham stimulation, an effect present in patients with higher impairment.

**Conclusion:**

tDCS of M1_affected hemisphere _can modulate performance of motor tasks simpler than those previously studied, a finding that could potentially benefit patients with relatively higher impairment levels.

## Background

Rehabilitative treatments result in incomplete motor recovery after stroke. Six months after stroke, 65 percent of survivors cannot use their paretic hand into daily living activities to the extent done before [1-5]. Absence or minimal hand movements by four weeks is predictive of poor motor outcome [6] and only a small percentage of patients recover function to the extent of community-matched healthy subjects [7] resulting in marked difficulties in carrying out activities of daily living (ADL).

Recent studies demonstrated that enhancing activity in motor areas of the affected hemisphere (M1_affected hemisphere_) by means of anodal transcranial DC stimulation (tDCS) [8-10] or repetitive transcranial magnetic stimulation (rTMS) [11] results in improvements in performance of complex ADL-like tasks with the paretic hand [12]. The tasks utilized in these studies, including the Jebsen-Taylor test (JTT), are cognitively demanding, only in part preprogrammed, and require complex online updating of sensory information as well as adequate sensorimotor integration for accurate performance [13]. Successful performance of the JTT requires a complex pattern of activation of muscles and joints as well as the use of targets and tools [13] and such skilled movements engage an extensive network of brain regions [14-16]. As a consequence, ADL-like tasks included in the JTT can be fully carried out by a minority of stroke patients [6].

At present, it is not known if the beneficial effects of tDCS apply only to complex motor tasks in patients with reduced impairment, capable of performing ADL-like tasks or if it can also benefit performance of simpler motor tasks which can be done by patients with higher impairment levels. In this investigation, we studied the effects of anodal tDCS on performance of motor tasks that predominantly rely on M1 functioning such as maximal pinch force production and a simple reaction time task [17-20]. We hypothesized that noninvasive anodal tDCS [8,21,22] applied to M1_affected hemisphere _would improve reaction time and pinch force compared to Sham stimulation [23] in a group of patients with subcortical stroke. Attention, fatigue and discomfort as possible confounds were evaluated using visual analogue scales [8,10,23-28].

## Results

### Psychophysical data

INTERVENTION_(tDCS, Sham) _did not influence Attention (tDCS: 6.99 ± 0.22; Sham: 6.62 ± 0.27), Fatigue (tDCS: 7.01 ± 0.20; Sham: 7.26 ± 0.22). Discomfort/pain was negligible and comparable in the tDCS (1.67 ± 0.14) and Sham sessions (1.20 ± 0.14, paired t-test, ns) on a scale of 1 (no discomfort) to 10 (maximal pain). None of the patients were able to distinguish between the tDCS and the Sham sessions.

### Effects of anodal tDCS of M1_affected hemisphere_ on reaction time

**Figure 1:**
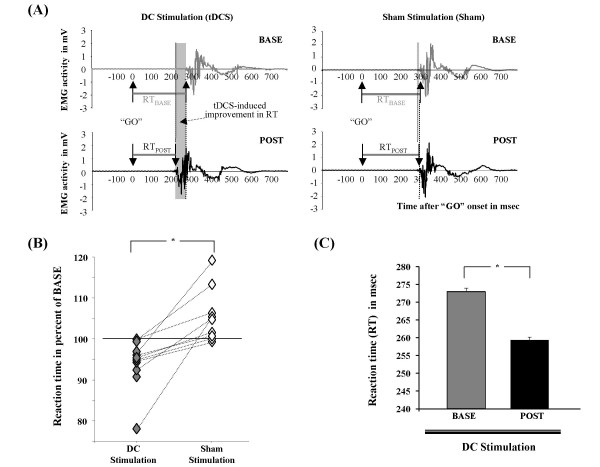
**(A) Effects of tDCS on RT (representative trials)**. RT, measured as the time between the GO-signal and the onset of EMG response, is shown at baseline (RT_Base_) and post intervention (RT_Post_) in representative trials in the tDCS (left) and Sham (right) sessions. Note the shorter RT_Post _than RT_Base _after tDCS but not Sham (highlighted in gray). X axis shows time (msec) and Y axis shows EMG activity (mV) **(B) Effects of tDCS on RT (individual subjects) **RT after tDCS and Sham relative to baseline (BASE) in all subjects (values > 100 indicate longer RT, whereas those < 100 indicate faster RT relative to baseline). Note that RT improved in all subjects with tDCS, while most subjects experienced longer RT with Sham, probably reflecting mild fatigue over the length of the experimental session (see Discussion). Group analysis showed that RT improvements were significantly different from Sham (paired t-test, p < 0.05), an effect present in all subjects (see individual subject connecting lines). **(C) Effects of tDCS on RT (group data) **tDCS shortened significantly RT (RT_Base_, paired t-test, p < 0.05). Y axis shows reaction times in msec.

The ANOVA_RM _revealed no significant effects of the factors INTERVENTION_(tDCS, Sham) _(F[1] = 0.02, ns) or TIME_(Base, Post) _(F[1] = 0.07, ns), but a significant interaction INTERVENTION_(tDCS, Sham) _× TIME_(Base, Post) _(F[1,10] = 11.9, p < 0.01; Fig. 1) on RT. Post hoc testing showed that RT experienced a significant reduction with tDCS (from Base_tDCS _= 273.5 ± 15.4 msec to Post_tDCS _= 256.6 ± 13.9 msec, p < 0.05; Fig. 1C) and a nonsignificant trend to lengthening with Sham (from Base_Sham _= 265.2 ± 10.7 msec to Post_Sham _= 277.7 ± 11.6 msec, p = 0.06). Baseline values did not differ significantly between Sham and tDCS. Given the wide inter-patient variability in reaction times (from 187 msec to 364 msec), post intervention values were also analyzed relative to baseline in each patient (Paired t-test, p < 0.05). All patients tested showed shortening of RT in the tDCS session (Fig. 1B). Six patients showed slightly longer reaction times with Sham compared to baseline.

**Figure 2:**
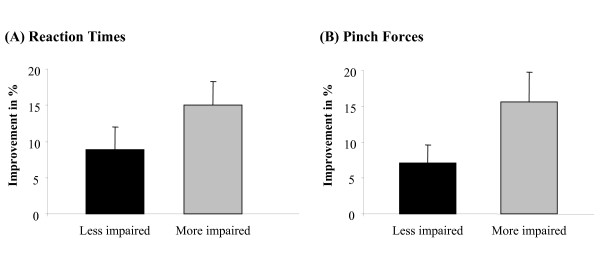
**Effects of tDCS and impairment of patients**. Patients were stratified in two groups according to their ability to perform skilled ADL-like motor tasks. Less impaired patients (n = 7) were able to perform the Jebsen-Taylor-Task (JTT) and more impaired patients were not able to perform the JTT. After stratification tDCS-induced improvement of reaction time **(A) **and pinch force **(B) **was calculated for each group. Note, that the improvement was larger in the more impaired group.

Stratification of patients according to impairment levels revealed that tDCS-induced improvement was more pronounced in the more impaired group (RT = 15.0 ± 3.2%; Fig. 2A) compared to the less impaired group (RT = 8.9 ± 3.1%; Fig. 2A). Correlation analyses showed a nonsignificant trend for more prominent tDCS-induced improvement in RT in patients with lower MRC scores (Spearman's rho [two tailed] R^2 ^= 0.36; p = 0.06)

### Effects of anodal tDCS of M1_affected hemisphere_ on pinch force

**Figure 3:**
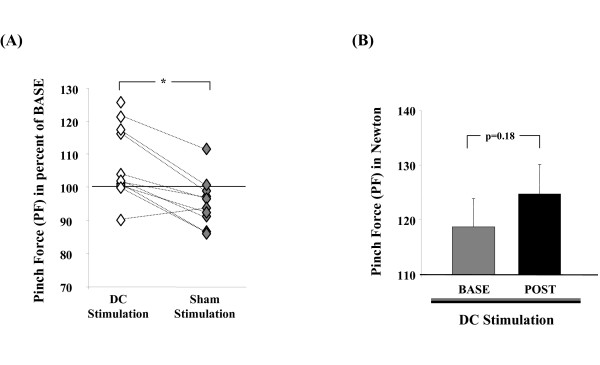
**(A) Effects of tDCS on PF (individual subjects)**. Pinch force after tDCS and Sham relative to baseline (BASE) in all subjects (values > 100 indicate stronger PF, whereas those < 100 indicate weaker PF relative to baseline). Note the significant improvement in PF after tDCS compared to Sham, present in all but one patient (paired t-test, p < 0.05, see also individual subject connecting lines). **(B) Effects of tDCS on PF (group data) **Group data showed a non-significant trend of increased pinch forces with tDCS (POST) compared to pinch forces during baseline (BASE; paired t-test, p = 0.18).

The ANOVA_RM _revealed no significant effects of the factors INTERVENTION_(tDCS , Sham) _(F[1] = 2.3, ns) or TIME_(Base, Post) _(F[1] = 0.03, ns), but a significant interaction INTERVENTION_(tDCS, Sham) _× TIME_(Base, Post) _(F[1,10] = 6.1, p < 0.05) on PF. Post hoc testing showed that tDCS elicited a nonsignificant trend towards increase in PF with tDCS (from Base_tDCS _= 118.8 ± 23.0 N to Post_tDCS _= 124.8 ± 24.0 N; p = 0.18) and towards decrease with Sham (from Base_Sham _= 114.8 ± 20.4 N to Post_Sham _= 111.2 ± 19.8 N; p = 0.20, Fig 3) with comparable baselines (p = 0.51). Given the wide inter-patient variability in absolute PF (from 22.1 N to 249.9 N), post intervention values were also expressed relative to baseline in each patient. All patients but one showed improvements in PF with tDCS relative to changes experienced with Sham (Fig. 3A, paired t-test p < 0.05). Six patients showed a transient slight decrease in force with Sham compared to baseline.

Stratification of patients according to impairment levels revealed that tDCS-induced improvement was more pronounced in the more impaired group (PF = 15.7 ± 4.1%; Fig. 2B) compared to the less impaired group (PF = 7.1 ± 2.5%; Fig. 2B). Correlation analyses did not show a significant correlation between tDCS-induced improvement in PF and the patients' MRC scores (Spearman's rho [two tailed] R^2 ^= 0.08; p = 0.27).

### Effects of Sham tDCS of M1_affected hemisphere_ compared to the no stimulation condition

**Figure 4:**
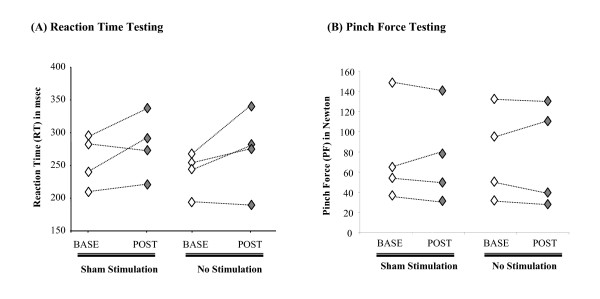
**(A) Effects of Sham stimulation on RT compared to No stimulation**. Reaction times were comparable during Sham stimulation and No stimulation with slight slowing of reaction times during POST compared to BASE. **(B) Effects of Sham stimulation on PF compared to No stimulation **Forces were comparable during Sham stimulation and No stimulation with slight decrease of pinch force during POST compared to BASE.

To determine if relatively slower RT and mild decrement in PF during Sham were a consequence of the 30 sec application of tDCS, we compared effects of Sham (30 sec tDCS, see Methods) with no stimulation (n = 4). Both Sham and no stimulation elicited comparable trends towards slightly longer RT and weaker PF (Fig. 4; RT: Base_No Stimulation _= 240.9 ± 16.0 N, Post_No Stimulation _= 272.6 ± 31.3 N and Base_Sham _= 257.2 ± 19.5 N, Post_Sham _= 281.3 ± 24.0 N; PF: Base_No Stimulation _= 75.8 ± 22.6 N, Post_No Stimulation _= 76.16 ± 25.1 N and Base_Sham _= 74.8 ± 25.0 N, Post_Sham _= 73.9 ± 23.9 N) consistent with the independence of these effects from the 30 sec tDCS application during Sham.

## Discussions and conclusion

The main finding of this double-blind sham-controlled cross-over study was that a 20 min period of noninvasive anodal tDCS applied to M1_affected hemisphere _resulted in transient improvements in maximal pinch force and reaction times of the paretic hand relative to sham in a group of chronic stroke patients in the absence of measurable nonspecific changes in attention, discomfort or fatigue. These effects were documented in every single patient tested with RT and in all but one with PF. All patients were blind to the intervention type as were investigators testing the endpoint measures.

Previous reports using tDCS and rTMS showed improvements in performance of tasks that mimic activities of daily living performed with the paretic hand after noninvasive cortical stimulation of M1_affected hemisphere _[8-11,29]. Performance of these tasks, like the Jebsen-Taylor test, require complex visuomotor integration and skilled coordination of force production as well as proper management of attentional and motivational resources [8,13]. This study, designed to determine if anodal tDCS over M1_affected hemisphere _exerted comparable effects over simpler motor tasks, identified improvements in pinch force and reaction times in the paretic hand with this intervention but not with Sham or No stimulation. These effects suggest that anodal tDCS to M1_affected hemisphere _enhanced activity within the primary motor cortex [8,22,30], actively engaged in control of force production and simple visuomotor integration processes [17,18,31-33].

The lack of effects on attention, fatigue and discomfort evidenced by the analysis of visual analog scales, as well as the inability of patients to identify the stimulation and Sham sessions are consistent with results from previous investigations [8,10,23-28]. Furthermore, tDCS was applied over the primary motor cortex, distant from areas involved in modulating attentional and motivational processes (e.g., cingulate cortex, prefrontal cortex, limbic system) [34,35]. Interestingly, repetition of both tasks after Sham resulted in a trend towards longer reaction times and weaker pinch force. The finding of this effect in the control experiment with both Sham (30 sec tDCS) and No stimulation (no tDCS) indicates that it was unrelated with the application of 30 sec tDCS during Sham. Most likely, it reflects mild experimental fatigue, subthreshold for detection by the VAS questionnaires [8,10].

Recovery of motor function after stroke starts first with improvements in performance of repetitive, relatively simple motor activities like force production, evolving later to relearning of more complex motor synergies and skillful tasks. It is a common finding that while most chronic stroke patients are able to generate various levels of force, only a fraction of them are able to perform skilled motor tasks like those involved in activities of daily living tested with the Jebsen-Taylor test [36]. Since the ability to control properly force production and visuomotor integration represents a prerequisite to meaningful skilled complex motor activity [37-39], it can be speculated that a first rehabilitative step to promote functional recovery after stroke might focus on reacquisition of these motor primaries. Subsequent training could then utilize these basic skills to train more complex actions, such as proposed in sports and musical practice programs [40]. In this way, motor training of these primaries could be utilized to orchestrate more complex skillful motor tasks [41-43]. The finding that anodal tDCS applied to M1_affected hemisphere _improved force production and a simple visuomotor integration task relative to Sham suggests that these mechanisms could contribute to the behavioral gains reported by anodal tDCS of M1_affected hemisphere _on more complex activities of daily living [8-11]. Of note is that these improvements were present in patients with higher impairment levels unable to perform skilled ADL-like motor tasks (Fig 2).

In summary, the present study provides novel evidence that anodal tDCS of M1_affected hemisphere _may enhance performance of a wider range of motor tasks than previously thought, some of them relatively simple and mediated predominantly by M1 function.

## Methods

### Patients

We studied eleven patients (57.0 ± 16.0 years; 5 of them females, 9 right-handed, 2 left-handed) with a history of a single mostly subcortical ischemic cerebral infarct, none of whom had a lesion in the primary motor cortex (Table 1). They gave written informed consent to participate in the experiment according to the declaration of Helsinki [44,45] and the NINDS Institutional Review Board approved the study protocol. Patients were tested at least 12 months after the stroke (41.8 ± 26.4 months, ranging from 18 to 107 months, Table 1). All patients suffered initially from a severe upper arm motor paresis (below MRC grade 2). Motor functions recovered over time and all patients were able to perform the required task properly while some of them remained unable to complete the Jebsen Taylor Test (JTT) test evaluated in previous experiments [8]. For detailed clinical information including Fugl-Meyer scale (FMS) [46], Medical Research Council scale (MRC), Modified Ashworth Scale for Grading Spasticity (ASS) [47] and Mini-Mental-Status-Examination (MMSE) [48] please see Table 1. Patients with severe language disturbances, history of severe alcohol or drug abuse, severe depression, or serious cognitive deficits (MMSE <23/30 points) were excluded from participation. One patient did only participate in the tDCS session and one patient did not perform the reaction time task.

### Experimental procedures

#### Main experiment

**Figure 5:**
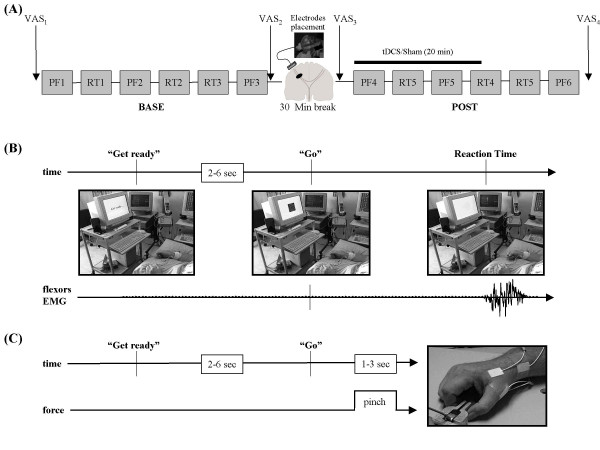
**(A) Experimental design of a single session**. In this study we used a double-blind, crossover study design with 2 sessions (tDCS and Sham). Half of the patients started with tDCS and the other half with Sham. Each session started with baseline determinations (BASE) of reaction times (RT_1–3_) and pinch force (PF_1–3_), followed by a 30 min break in which tDCS electrodes were placed. Then tDCS or Sham was applied in a counterbalanced double-blind design followed by post intervention measures (RT_4–6 _and PF_4–6_). All patients described their level of attention toward the task (range: 1–10; 1 = no attention, 10 = highest level of attention) and their perception of fatigue (range: 1–10; 1 = highest level of fatigue, 10 = no fatigue) four times in each session (VAS_1_-VAS_4_), and their sense of discomfort/pain after each session ended (range: 1–10; 1 = no discomfort/pain, 10 = maximal discomfort/pain) using visual analog scales (VAS) that have good internal consistency, reliability, and objectivity [24, 25, 27, 28]. Instructions to the patients were identical for all Sessions. In 4 patients an additional session was performed as a control experiment to evaluate the effects of No Stimulation compared to Sham stimulation. **(B) Reaction Time Testing during a Visuo-Motor Task **Patients were seated in a comfortable armchair and were instructed to focus attention on a cross in the centre of a video screen, and to bend their wrist as quickly as possible in response to a GO-signal presented on the screen. Trials were started with a visual warning signal ('Get ready'), followed by a GO-signal at random intervals (2–6 seconds). Blocks consisted of 23 wrist flexion trials with the first three trials of a block used as practice trials. These trials were not included in the analysis. EMG was recorded from silver-silver chloride electrodes positioned in a belly tendon montage on the skin overlying the Flexor Carpi Radialis muscle. Reaction times (RT) were defined as the time interval between the GO-signal and the onset of the EMG-burst in the Flexor Carpi Radialis muscle. Patients didn't receive any feedback about there performance. **(C) Pinch Force Testing Task **Patients were seated in a comfortable armchair with both arms relaxed. Maximal pinch strength of the paretic hand was measured according to a protocol with good validity and test-retest reliability [49, 50]. Patients held the arm of a dynamometer between the lateral aspect of the middle phalanx of the index finger and the thumb pad. Trials were started with a warning signal ('Get ready'), followed by a GO-signal at random intervals (2–6 seconds). Patients were instructed to squeeze the gauge as hard as they could for 1–3 seconds after GO-signal. Patients didn't receive any feedback about there performance. Blocks consisted of nine consecutive trials.

We tested the effects of anodal tDCS applied to M1_affected hemisphere _on performance of a simple reaction time task (RT) and on pinch force production (PF) in a pseudo-randomized, double-blind Sham-controlled cross-over study design. All patients participated in two sessions (tDCS and Sham separated by 8.2 ± 1.5 days (mean ± SE). Before starting the first session patients got familiarized with the tasks. Half of them started with tDCS and the other half with Sham. Patients performed three blocks of the RT task and three blocks of the PF task before (RT_1 _to RT_3 _and PF_1 _to PF_3_) and after (RT_4 _to RT_6_, PF_4 _to PF_6_) anodal tDCS and Sham applied to M1_affected hemisphere_. The last measurements were recorded 29.7 ± 4.2 min after the end of each intervention. Order of tasks was balanced over subjects and sessions. Instructions to the patients were identical for both Sessions (tDCS and Sham). Questionnaires using visual analog scales (VAS) to evaluate patients' perception of attention (range: 1–10; 1 = no attention, 10 = highest level of attention) and fatigue (range: 1–10; 1 = highest level of fatigue, 10 = no fatigue) were determined two times before and two times after tDCS and Sham (see VAS1-4 in Fig 5A). At the end of each session patients were asked to describe their sense of discomfort/pain (range: 1–10; 1 = no discomfort/pain, 10 = maximal discomfort/pain). The used VAS have good internal consistency, reliability, and objectivity [24-28]. After the end of the study the investigators asked the patients whether they could identify the tDCS and the Sham sessions. All patients but one participated in Session 1 and Session 2. Figure 5A displays a schematic of the experimental design.

In an additional session (control experiment), we evaluated the effects of no stimulation (no tDCS, n = 4) and compared the results with those obtained with Sham. In all sessions patients had the scalp electrodes placed in position and the sessions had a comparable time course.

#### Reaction time testing

Patients were seated 60 cm in front of a 20 inch-monitor with both arms supported by a cushion. They were instructed to focus on the cross in the centre of the screen, and to bend their wrist as quickly as possible in response to the GO-signal (Fig. 5B). Each trial started with a visual warning signal ('Get ready...'), which was followed by a GO-signal at random intervals (2–6 seconds). Each block consisted of 23 wrist flexion trials. The first three trials of every block were used as practice trials and were not included in the analysis. Electromyogram (EMG) was recorded from silver-silver chloride electrodes positioned in a belly tendon montage on the skin overlying the Flexor Carpi Radialis muscle (50 Hz-2 kHz, sampling rate 5 kHz) from a Counterpoint Electromyograph (Dantec Electronics Sklovlunde, Denmark). The reaction time (RT in msec) was defined as the time interval between the GO-signal and the onset of the EMG-burst (Fig. 5B, 1A). Patients didn't receive any feedback about their performance. Reaction times were analyzed by an investigator blinded towards the INTERVENTION type (Sham, tDCS) and towards TIME (Base, Post). Trials with EMG activity in the rest period before the GO-signal were excluded from analysis.

#### Pinch force testing

Patients were seated in an armchair with both arms relaxed. Maximal pinch force of the paretic hand was measured according to a protocol with good validity and test-retest reliability [49,50]. Patients held the arm of a dynamometer between the lateral aspect of the middle phalanx of the index finger and the thumb pad of the paretic hand and were instructed to squeeze the gauge as hard as they could for 1–3 seconds (Fig. 4C). Patients didn't receive any feedback about their performance. Nine muscle strength measurements were averaged in each block. Maximal pinch force (PF) was elicited by pinching the gauge between the thumb and the index finger of the paretic hand. Trials in which pinch force was produced during the rest period where excluded from analysis.

#### Noninvasive anodal tDCS

Anodal tDCS was delivered for 20 minutes in the tDCS session and for up to 30 seconds in the Sham session using a Phoresor^® ^II Auto (Model No. PM850, IOMED^®^, Salt Lake City, Utah 84120 USA) through two gel-sponge electrodes (TransQE from IOMED^®^, surface area was 25 cm^2 ^for each electrode) embedded in a saline-soaked solution. For both interventions (tDCS and Sham) current was increased in a ramp-like fashion at the onset of the stimulation [8,21] eliciting a transient tingling, burning sensation on the scalp that disappeared over seconds, consistent with previous reports [8,21,23]. Current (1 mA) remained on for 20 minutes in the tDCS session and for up to 30 seconds in the Sham session. At the end of the session, tDCS was turned off slowly over a few seconds, a procedure that does not elicit additional perceptions [8,21].

#### Blinding procedure

The patients and the investigator performing motor testing and data analysis were blinded to the type of intervention (tDCS or Sham) as described previously [8,10,23]. The tDCS device remained out of the patients' view at all times. Another unblinded investigator's sole participation in the study consisted in administering the interventions. Participants were explicitly asked after the experiments whether they are able to determine which of the experimental session was "real" stimulation and which one was "not real, sham" stimulation.

#### Electrode positions

In seven patients, the anode was positioned on the projection of the hand knob area [51] of the primary motor cortex of the affected hemisphere on the patient's scalp by using coregistered MRI for neuronavigation (Brainsight^®^). In 4 patients in whom MRI was not available at the time of testing, the anode was centered on the optimal scalp position for activation of the paretic First Dorsal Interosseus (FDI) muscle using an 8-shaped TMS Magstim coil, a procedure that matches the projection of the anatomical hand knob [52]. The cathode was placed on the skin overlying the contralateral supraorbital region.

### Data analysis

Reaction time and pinch force data were normally distributed as evaluated by Kolmogorov-Smirnov test. For statistical analysis the three baseline (RT_1 _to RT_3_, PF_1 _to PF_3_) and the three post intervention measurements (RT_4 _to RT_6_, PF_4 _to PF_6_) were pooled (Fig 5A). Repeated measures ANOVA (ANOVA_RM_) was used to evaluate the effects of INTERVENTION_(tDCS, Sham) _and TIME_(Base, Post) _on RT and on PF. Paired t-tests were used to evaluate reports of discomfort/pain. For the control experiment paired t-tests were used to compare the No stimulation with the Sham condition. Non-parametric Friedman test was used to evaluate the effects of INTERVENTION_(tDCS, Sham) _on attention and fatigue, which were not normally distributed. Conditioned on significant p-values (p < 0.05), post-hoc testing was performed and corrected for multiple comparisons when necessary. All data are expressed as mean ± SE.

## Abbreviations

ADL = Activities of daily living

ASS = Modified Ashworth Scale for Grading Spasticity

Base_tDCS _= Baseline before tDCS as intervention

Base_Sham _= Baseline before Sham as intervention

Base_No Stimulation _= Baseline before No Stimulation as intervention

FMS = Fugl-Meyer scale

JTT = Jebsen-Taylor Hand Function Test

M1 = Primary motor cortex

M1_affected hemisphere _= Motor cortex of the affected hemisphere

MMSE = Mini-Mental-Status-Examination

MRC = Medical Research Council scale

PF = Pinch force

PFT = Pinch force testing

Post_tDCS _= After tDCS as intervention

Post_Sham _= After Sham as intervention

Post_No Stimulation _= After No Stimulation as intervention

RT = Reaction time

rTMS = Repetitive transcranial magnetic stimulation

RTT = Reaction time testing

tDCS = Transcranial DC stimulation

VAS = Visual analog scale

## Competing interests

The author(s) declare that they have no competing interests.

## Authors' contributions

FH, LC, CG, PC, AF, PG participated in the design of the study; FH, PG, PC, AF, BV participated in acquisition and analysis of data; all authors participated in the interpretation of the data; FH, LC drafted the manuscript; all authors read and approved the manuscript.

Part of the data from one of these patients has been published as a case report.

## Figures and Tables

**Table 1:** 

**Patient**	**Age**	**Sex**	**Time after stroke (mo)**	**Handedness (EDS)**	**MMSE**	**Motor Function**		
						
						**MRC**	**FMS**	**ASS**
A	35	f	72	Right (48/50)	30/30	4.0	.85	1
B	41	m	48	Left (12/50)	30/30	4.80	.95	2
C	72	f	34	Right (46/50)	29/30	4.80	.96	0
D	44	f	24	Right (48/50)	29/30	4.90	.99	0
E	38	f	31	Right (49/50)	29/30	4.90	.99	0
F	61	m	42	Right (49/50)	28/30	4.50	.79	3
G	72	m	23	Right (46/50)	30/30	4.70	.91	1+
H	61	f	38	Right (42/50)	29/30	3.80	.82	3
I	66	m	23	Right (49/50)	28/30	4.90	.95	0
J	54	m	18	Right (46/50)	28/30	4	.81	2
K	84	m	107	Right (47/50)	29/30	4.80	.96	0

ASS = Ashworth Spasticity Score; EDS = Edinburgh-Handedness-Scale; FMS = Fugl-Meyer-Scale (presented in relative terms of the maximum of points [66]); MMSE = Mini-Mental-Status-Examination; MRC = Scale to determine strength by the Medical Research Council (mean MRC value of the tested muscles).
